# Supplementary material for: Tomographic docking suggests the mechanism of auxin receptor TIR1 selectivity
Source: Open Biol. 2016 Oct 19;6(10):160139. doi: 10.1098/rsob.160139 (PMC5090058; doi:10.1098/rsob.160139)
Supplement: Supplementary Material [file rsob160139supp1.pdf]

Tomographic docking suggests the mechanism of auxin receptor TIR1 selectivity
Supplementary material

Veselina V. Uzunova, Mussa Quareshy, Charo I. del Genio, and Richard M. Napier
School of Life Sciences, University of Warwick, Gibbet Hill Road, Coventry CV4 7AL, UK
(Dated: August 28, 2016)

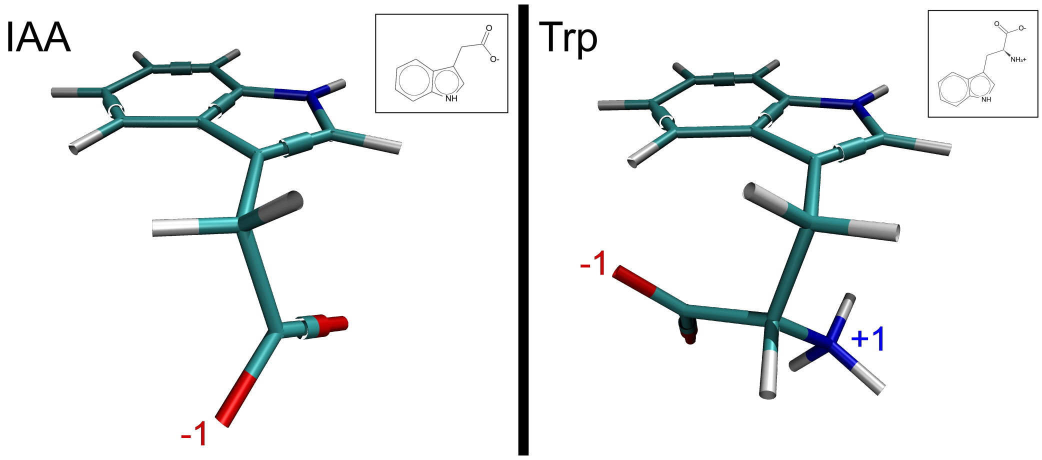

Figure S1: Structures of IAA (on the left) and Trp (on the right), displaying bond orders and charges at pH=7.3. The insets show the respective skeletal structural formulae.

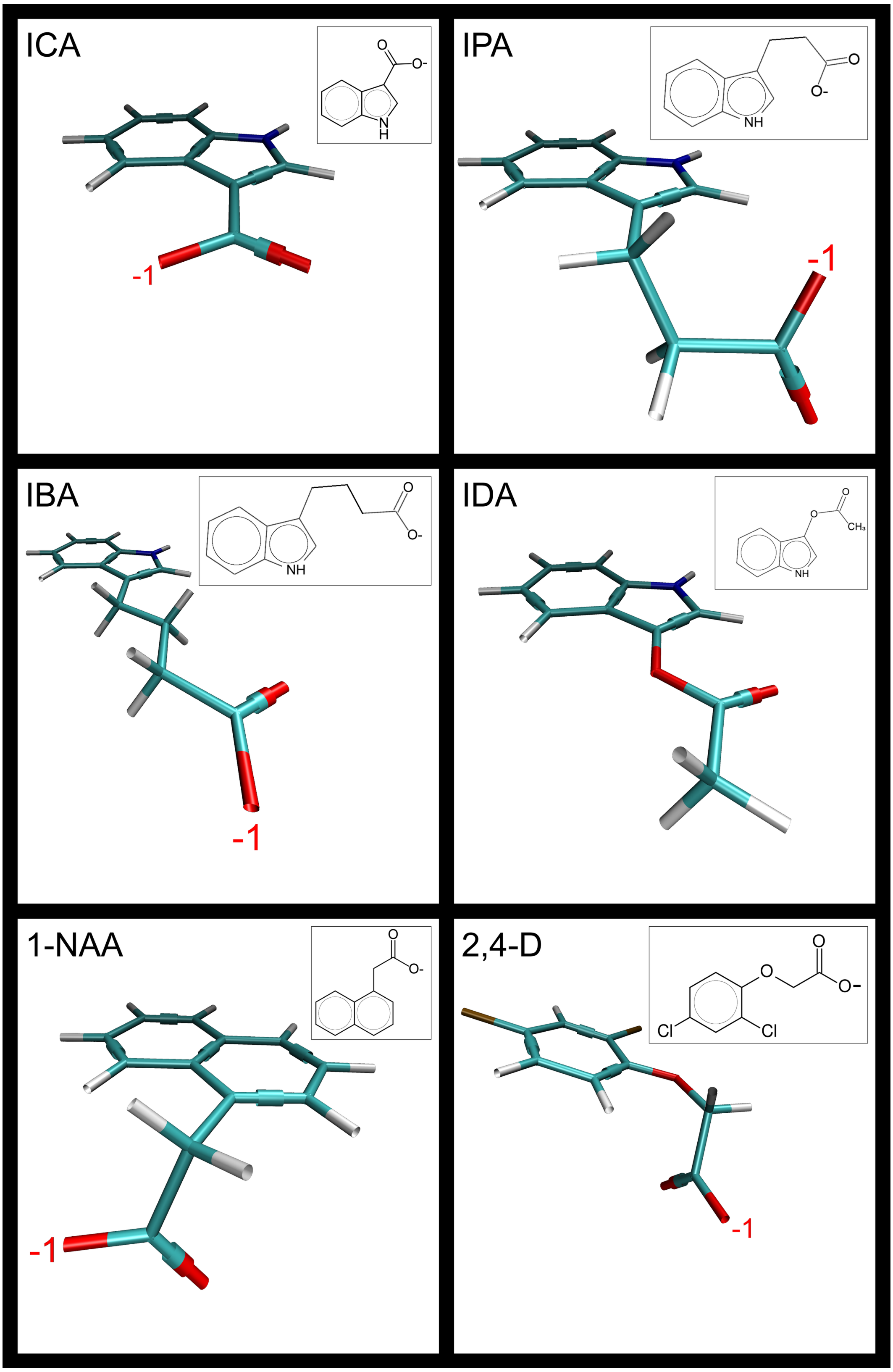

Figure S2: Structure of compounds used for validation. All representations show bond order and charges at pH=7.3, with skeletal structural formulae in the insets. Top-left: indole-3-carboxylic acid (ICA). Top-right: indole-3-propionic acid (IPA). Middle-left: indole-3-butyric acid (IBA). Middle-right: 3-indolyl acetate (IDA). Bottom-left: 1-naphthaleneacetic acid (1-NAA). Bottom-right: 2,4-dichlorophenoxyacetic acid (2,4-D).

Residue	Atoms in search space at step number														
	1	2	3	4	5	6	7	8	9	10	11	12	13	14	15
HIS-78	0	0	0	0	0	1	1	3	6	6	6	6	6	6	6
PHE-79	0	1	1	3	4	4	4	4	4	4	4	4	4	4	4
PHE-82	1	3	4	6	7	9	9	9	9	9	9	9	9	9	9
LEU-84	5	5	5	5	5	5	5	5	5	5	5	5	5	5	5
VAL-321	0	0	0	0	0	0	0	0	0	0	0	0	0	0	1
ARG-344	0	0	0	0	0	0	0	0	0	0	0	0	0	0	2
VAL-345	0	0	0	0	0	0	0	0	0	0	0	0	1	2	6
PHE-346	0	0	0	0	0	0	0	0	0	0	1	5	8	11	12
PRO-347	0	0	0	0	0	0	1	3	5	6	6	6	6	6	6
PRO-350	1	2	3	6	6	6	6	6	6	6	6	6	6	6	6
PHE-351	5	5	5	5	5	5	5	5	5	5	5	5	5	5	5
MET-353	2	2	2	2	2	2	2	2	2	2	2	2	2	2	2
GLU-361	0	0	0	0	0	0	0	0	0	0	0	0	0	1	1
LEU-378	0	0	0	0	0	0	0	0	0	0	0	1	3	3	7
TYR-379	0	0	0	0	0	0	0	0	0	0	0	2	3	7	10
PHE-380	0	0	0	0	0	0	0	2	4	9	10	12	12	12	12
CYS-381	0	0	0	0	0	0	1	2	4	5	7	7	7	7	7
GLN-383	0	0	0	0	0	0	0	0	1	2	2	2	2	2	2
MET-384	0	0	0	0	0	0	0	0	1	2	2	2	4	4	4
PHE-402	0	0	0	0	0	0	0	0	0	0	0	0	0	1	4
ARG-403	0	0	0	0	0	0	0	0	0	0	1	4	10	14	17
LEU-404	0	0	0	0	0	0	0	0	1	2	6	9	9	9	9
CYS-405	0	0	0	0	0	1	***1***	***5***	5	7	7	7	7	7	7
ILE-406	0	0	0	1	2	5	7	9	9	9	9	9	9	9	9
ILE-407	0	2	3	5	8	9	9	9	9	9	9	9	9	9	9
GLU-408	7	9	10	10	10	10	10	10	10	10	10	10	10	10	10
PRO-409	7	7	7	7	7	7	7	7	7	7	7	7	7	7	7
LYS-410	10	10	10	10	10	10	10	10	10	10	10	8	7	6	5
ALA-411	1	3	4	4	4	4	4	4	4	4	4	4	4	4	4
PHE-424	0	0	0	0	0	0	0	0	0	2	3	4	4	4	4
ARG-436	0	0	0	0	0	0	0	0	0	0	1	4	6	9	12
LEU-437	0	0	0	0	0	0	0	0	0	0	1	3	7	8	8
SER-438	0	0	0	0	0	0	0	0	***0***	***5***	6	8	8	8	8
LEU-439	0	0	0	0	0	***1***	***4***	***7***	***9***	9	9	9	9	9	9
SER-440	0	0	0	0	0	3	7	8	8	8	8	8	8	8	8
GLY-441	0	0	1	3	4	4	4	4	4	4	4	4	4	4	4
SER-462	0	0	0	0	0	***0***	***2***	***3***	***5***	5	5	5	5	5	5
VAL-463	0	0	2	3	5	5	5	5	5	5	5	5	5	5	5
ALA-464	1	4	5	6	6	6	6	6	6	6	6	6	6	6	6
PHE-465	12	12	12	12	12	12	12	12	12	12	12	12	12	11	9
ALA-466	4	5	5	5	5	5	5	5	5	5	5	5	5	5	5
ARG-489	***9***	***13***	***14***	***16***	16	16	16	16	16	16	16	16	16	16	16
ASP-490	9	9	9	9	9	9	9	9	9	9	9	9	8	5	0
CYS-491	3	3	3	3	3	3	3	3	3	3	3	3	3	3	1
PRO-492	1	1	1	1	1	1	1	1	1	1	1	1	1	1	0
SER-515	3	3	3	3	3	3	3	3	3	3	1	0	0	0	0
IHP-601	0	0	0	0	0	0	0	0	0	0	0	0	1	1	2

Table S1: Number of atoms belonging to each residue included in the search space at each step. The residues in violet are those belonging to the engagement niche; those in orange constitute the molecular filter. The steps across which the contribution of the molecular filter residues changes significantly are highlighted in bold italic typefont.
